# Pilot study of locomotion improvement using hybrid assistive limb in chronic stroke patients

**DOI:** 10.1186/1471-2377-13-141

**Published:** 2013-10-07

**Authors:** Hiroaki Kawamoto, Kiyotaka Kamibayashi, Yoshio Nakata, Kanako Yamawaki, Ryohei Ariyasu, Yoshiyuki Sankai, Masataka Sakane, Kiyoshi Eguchi, Naoyuki Ochiai

**Affiliations:** 1 Faculty of Engineering, Information and Systems, University of Tsukuba, Ibaraki, Japan; 2 Faculty of Medicine, University of Tsukuba, Ibaraki, Japan; 3 Tsukuba Critical Path Research and Education Integrated Leading Center (CREIL), University of Tsukuba, Ibaraki, Japan; 4 Center for Cybernics Research (CCR), University of Tsukuba, Ibaraki, Japan

## Abstract

**Background:**

Locomotor training using robots is increasingly being used for rehabilitation to reduce manpower and the heavy burden on therapists, and the effectiveness of such techniques has been investigated. The robot suit Hybrid Assistive Limb (HAL) has been developed to rehabilitate or support motor function in people with disabilities. The HAL provides motion support that is tailored to the wearer’s voluntary drive. We performed a pilot clinical trial to investigate the feasibility of locomotor training using the HAL in chronic stroke patients, and to examine differences between two functional ambulation subgroups.

**Methods:**

Sixteen stroke patients in the chronic stage participated in this study. All patients were trained with the HAL over 16 sessions (20–30 min/day within 2 days/week). Primary outcomes were walking speed, cadence, and number of steps recorded during a 10-meter walk test (10MWT). Berg balance scale (BBS) and timed up and go (TUG) were also measured. All outcome measures were evaluated without wearing HAL assistance before and after the intervention in all patients as well as in the dependent ambulatory and independent ambulatory subgroups.

**Results:**

All participants completed the intervention with no adverse events. Gait speed, cadence, number of steps during the 10MWT, and BBS increased significantly from 0.41 m/s to 0.45 m/s (P = 0.031), from 68.6 steps/min to 72.0 steps/min (P = 0.020), from 37.5 steps to 33.1 steps (P = 0.017), and from 40.6 to 45.4 (P = 0.004) respectively. The TUG test score improved, although this difference was not statistically significant. The findings in the dependent ambulatory subgroup primarily contributed to the significant differences observed in the group as a whole.

**Conclusions:**

This pilot study showed that locomotor training using the HAL is feasible for chronic stroke patients. Randomized controlled trials are now required to demonstrate the effectiveness of HAL-based rehabilitation over conventional therapies.

**Trial registration:**

UMIN000002969

## Background

Stroke is the second largest cause of death in the world [1] and is a major cause of paralysis and other physical or cognitive disabilities [2-4]. Patients with impaired walking ability caused by lower-limb paralysis often become dependent on a wheelchair or may even be bedridden [5]. Therefore, restoration of walking ability is extremely important for maintaining or regaining quality of life, as well as activities of daily living and social reintegration.

Task-specific training programs that improve the motor function by motor learning of a repeatedly executing motor task are increasingly being adopted as advanced therapy for central nervous system diseases, including stroke [6]. Locomotor training based on gait motion has been suggested for task-specific training programs aimed at restoring walking ability [7-9]. Indeed, body weight-supported treadmill training is widely used in clinical research [10-12]. Partial body weight support makes it easier to maintain an upright posture by reducing the weight load on the lower limbs. Swinging the legs with manual assistance from the therapists can also allow patients with significant deficits to perform repetitive gait motions. However, this training method requires extensive manpower and places a heavy burden on the therapists. In such procedures, one or two therapists are positioned on one side or both sides of the treadmill to manually assist leg motion, which also causes fatigues in the therapist.

To overcome this limitation, robots have been developed to support gait motion. Compared with therapists, robots are better able to provide cyclic support of the patient’s leg motion: in therapists, excessive fatigue is imposed because of repeated manual support that demands a significant amount of energy [13]. The therapists, who are now released from that heavy burden, can then give the patients valuable advice and/or guidance for locomotor training.

So far, two types of robots have been developed for gait training. The first type involves an exoskeleton (e.g. Lokomat [14], LOPES, ALEX [15], and AutoAmbulator [16]), which has leg joints that match those of the patient’s legs and are positioned next to them. Actuators placed at the joints of the robot control joint motions to mimic normal walking patterns, and allow the patient’s joints to move in synchronization with the robot’s motion. The second type of robot is the end-effector type (e.g., Gait Trainer [17], Haptic Walker [18], and LokoHelp [19]), in which only the patient’s soles are fixed to the robot’s foot plates. With these robots, the motion of the robot’s foot plates mimics normal gait and guides the patient’s feet.

Several clinical trials of robot-assisted gait training have been conducted in stroke patients. Randomized controlled studies for the Lokomat [20-25] and the Gait Trainer [26-29] showed various degrees of effectiveness of gait restoration compared with therapist-assisted gait training or conventional therapy. Pilot clinical trials for the AutoAmbulator [16] and the LokoHelp [19] have also been conducted. These robots provide autonomous motion to patients based on a desired kinematic trajectory of the lower limb joints or the end effector, mimicking the walking motion of an able-bodied person [14,17]. To date, however, robot-based rehabilitation of walking has concentrated on locomotor training using the autonomous motion generated by the robots.

We developed the Hybrid Assistive Limb (HAL), a wearable robot that interactively provides motion according to the wearer’s voluntary drive [30]. The HAL detects the bioelectric signals generated by patient’s muscle activities and/or the floor reaction force signals caused by patient’s intended weight shifts. The HAL enables locomotor training with voluntary drive. HAL has the advantage of voluntary drive and ambulatory performance. The other exoskeletons use autonomously generated predefined motion for users. In contrast, HAL generates motion according to the wearer’s voluntary drive. The wearer operates the HAL by adjusting his/her muscle activities. Therefore, the HAL is able to conduct locomotor training by providing motion support in response to the user’s voluntary drive. This assistance mechanism is completely different from those of other exoskeletons. In addition, the other exoskeletons are designed for walking on a treadmill; therefore, they provide a simulated gait that differs from that of walking on a flat floor. In contrast, as a wearable system, the HAL delivers locomotor training in actual ambulatory environment. Kubot et al. reported that for patients with limited mobility including chronic stroke, gait speed increased after gait training with the HAL [31]. In this study, gait speed increases were significant for nine patients with chronic stroke. However, the feasibility of HAL-based training for improving walking ability or balance, and its benefits for patients with chronic stroke are unclear; therefore, preliminary data are needed before conducting randomized controlled trials to confirm its effectiveness. We conducted a pilot clinical trial to investigate the feasibility of locomotor training with the HAL in chronic stroke patients and to examine differences between two functional ambulation subgroups.

## Methods

### Patients

Sixteen stroke patients with hemiplegia (12 men and four women) participated in this study (Table 1). The mean ± standard deviation (SD) age at the time of study enrolment was 61.0 ± 14.8 years. All patients were in the chronic phase (time since stroke: 47.1 ± 37.6 months) enrolling in the study > 6 months after the first stroke and currently enrolled in physical therapy. The causes of stroke were hemorrhage (n = 12), ischemia (n = 2), ischemia and subarachnoid hemorrhage (n = 1), and moyamoya disease (n = 1). Seven patients had left-sided paralysis, while nine had right-side paralysis. Twelve were classified as Brunnstrom stages III, while the others were characterized as I, II, IV, and V (one each). Before the intervention, ten patients used a T-cane to walk, three used a quad-cane, and one used a pick-up walker. Twelve wore ankle foot orthoses, while one wore a knee ankle foot orthosis. Four patients were able to walk independently, five needed supervision during walking, and one was unable to walk. The mean Barthel index was 83.8 ± 15.0. All patients except for Cases 7, 8, and 15, underwent conventional rehabilitation or exercise instruction before this study. We divided the patients into two subgroups based on Functional Ambulation Category (FAC): a dependent ambulatory subgroup, in which eight patients required assistance from another person in the form of intermittent or continuous light touch to assist balance or coordination (FAC 2), or stand-by guarding or verbal cueing (FAC 3), and an independent ambulatory subgroup in which eight patients could walk freely on level surfaces only (FAC 4) or everywhere independently (FAC 5).

**Table 1:** Patient characteristics

**Case**	**Sex**	**Age**	**Time since stroke, months**	**Etiology**	**Side of paralysis**	**Br. stage**	**Assistive device**	**Orthosis**	**FAC**	**BI**	**Group**
1	M	65	26	Hemorrhage	R	III	Quad-cane	AFO	3	50	A
2	M	72	33	Ischemia	L	III	T-cane	AFO	3	75	A
3	M	54	13	Hemorrhage	L	III	T-cane	NA	3	80	A
4	F	63	27	Hemorrhage	R	III	T-cane	AFO	3	90	A
5	M	18	132	Moyamoya disease	L	IV	NA	AFO	5	100	B
6	M	74	42	Ischemia	L	III	T-cane	AFO	4	100	B
7	M	53	24	Ischemia, Subarachnoid hemorrhage	R	I	Pick-up walker	KAFO	2	65	A
8	M	64	13	Hemorrhage	R	III	T-cane	AFO	3	80	A
9	F	67	40	Hemorrhage	R	V	T-cane	NA	5	90	B
10	F	64	84	Hemorrhage	L	III	T-cane	AFO	4	70	B
11	M	61	40	Hemorrhage	L	III	NA	NA	4	85	B
12	M	67	48	Hemorrhage	R	III	Quad-cane	AFO	3	70	A
13	F	45	18	Hemorrhage	R	III	Quad-cane	AFO	4	85	B
14	M	84	56	Hemorrhage	R	III	T-cane	AFO	4	100	B
15	M	55	25	Hemorrhage	L	III	T-cane	AFO	4	100	B
16	M	70	132	Hemorrhage	R	II	T-cane	AFO	3	100	A

*M* male, *F* female, *L* left, *R* right, *Br*. Brunnstrom, *AFO* ankle foot orthosis, *KAFO* knee ankle foot orthosis, *NA* not applicable, *FAC* functional ambulation categories, *BI* Barthel index, *Group A* dependent ambulatory, *Group B* independent ambulatory.

The inclusion criteria were as follows: requirement of physical assistance or assistive devices for standing up, sitting down, and/or walking; understanding an explanation of the study protocol and expressing voluntary consent or refusal; a body shape that could fit in the robotic suit HAL (height, 150–180 cm; weight ≤80 kg); and concurrent use of physical and occupational therapies. Exclusion criteria were uncontrolled cardiorespiratory conditions, severe cognitive deficits affecting the ability to understand verbal instructions, severe contractures limiting the range of motion of the lower limb (loss of hip or knee extension >20°), and severe spasticity (Modified Ashworth Scale score >3). All patients provided written informed consent, including allowing the publication of individual clinical details, before participating in the study, which was approved by the institutional review board at the University of Tsukuba. This study was part of a research project whose protocol was registered with the UMIN Clinical Trials Registry (UMIN000002969).

### Intervention

**Figure 1:**
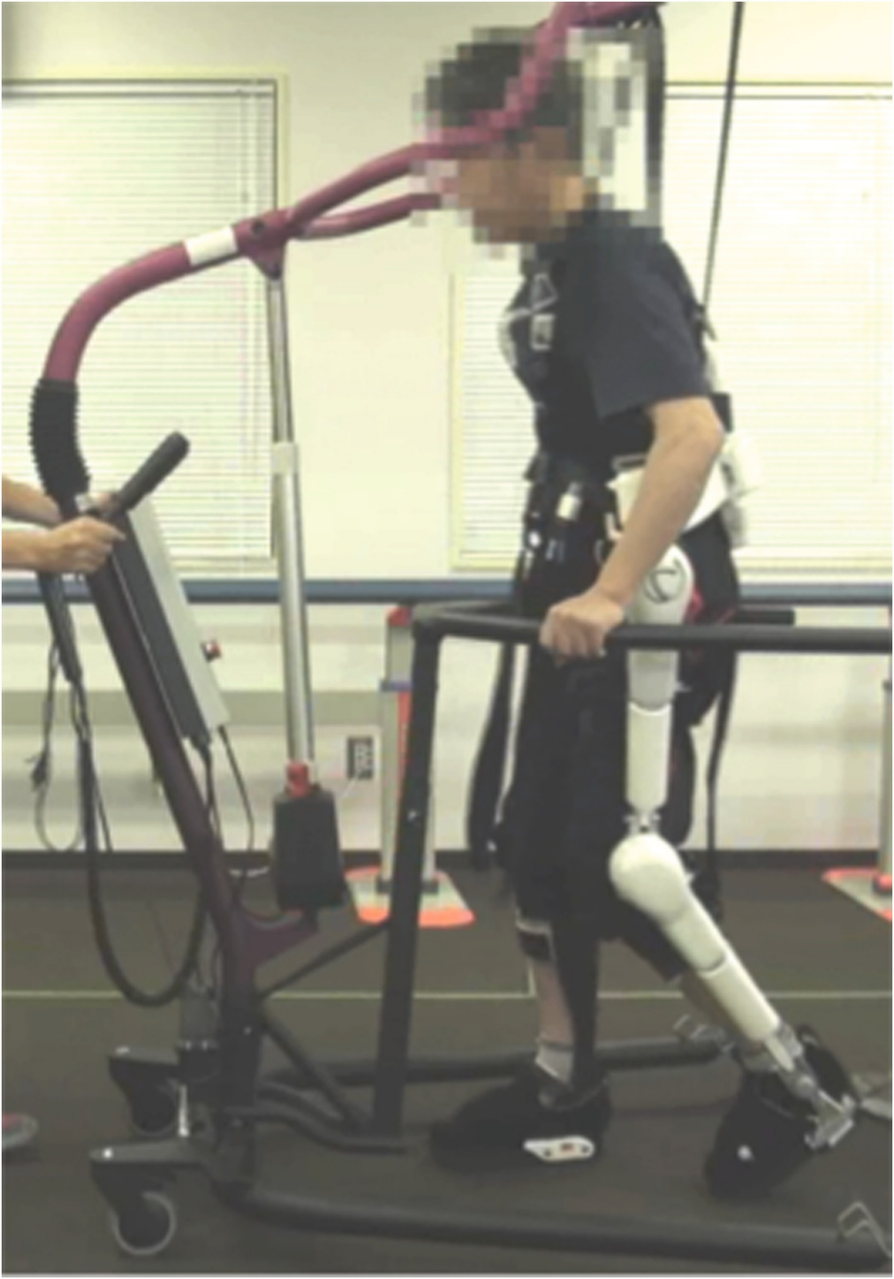
Photograph showing the Hybrid Assistive Limb (HAL) worn by a patient and the All-In-One Walking Trainer suspending the patient during the locomotor training.

All patients underwent 16 locomotor training sessions using the HAL within 2 days/week as tolerated by each patient. Each session lasted approximately 90 min, including setup of the HAL, rest periods, and assessments. The total duration of training with the HAL was between 20 and 30 min in each session. During locomotor training, the patients walked on a floor with robotic assistance from the HAL. To prevent falling, the patients wore a harness connected to a mobile suspension system (All-In-One Walking Trainer, Ropox A/S, Denmark) (Figure 1). The level of suspension was independently adjusted for each patient and increased as tolerated without excessive knee flexion during the stance phase or toe dragging during the swing phase. A therapist operated the suspension system to determine the walking speed and direction. Another therapist was positioned behind the patient to support hip and trunk stability if needed. As shown in Figure 1, the patients were allowed to hold onto the side rails of the suspension system to maintain their posture.

Early training sessions for individuals with severe gait impairment involved simple flexion/extension movements of the lower limb and dynamic postural tasks (i.e., sit-to-stand task) with HAL assistance to become familiar with operating HAL and receiving its assistance before progressing to walking training with it. The walking speed and distance during training were determined based on the patient’s tolerance. As the session progressed, the training intensity was gradually increased by changing the walking speed, duration of walking, and the degree of the body-weight unloading. The therapists provided verbal encouragement and advice to the patients regarding walking pattern and posture. Blood pressure and heart rate were measured at the start and end of each training session, and during the rest periods. At the end of each session, the patients stated their fatigue level after the training as well as their level of satisfaction with the robotic assistance during movement by using a visual analog scales.

During each training session, walking was continuously assisted by the HAL, a computer-controlled exoskeletal device. The design and control system for the HAL are described in more detail elsewhere [30]. Briefly, the exoskeletal frame was secured to the patients at the pelvis and at the lower limbs by cuffs. The joints of the HAL frame were aligned to the patient’s joints. Active joints-mounted actuators generated assistive torque at the hip and knee joints. The HAL has a hybrid control system consisting of a Cybernic Voluntary Control (CVC) and a Cybernic Autonomous Control (CAC) [30]. The CVC drives the amount and timing of the assistive torque provided to each joint for walking based on bioelectrical signals detected from skin surface electrodes placed over the flexor and extensor muscles of the hip and knee [32]. The gain in assistive torque at each joint in response to the bioelectrical signals was controlled by a therapist. The optimal gain and balance of the torque to maintain an appropriate walking pattern were determined by observing the joint trajectories and patient’s comments. If it was difficult for patients to achieve the motion derived from the CVC, the CAC can autonomously generate torque according to the walking pattern by referring to information from the floor reaction force [33] and was used until the patients became familiar with the CVC.

### Outcome measures

Outcome measures were collected for each participant before and after the HAL locomotor training. As a primary outcome measure, walking speed was assessed by the 10-meter walk test (10MWT), in which gait speed is sensitive to changes in locomotor recovery in chronic stroke patients [34,35]. The number of steps and cadence during the 10MWT were also assessed. Gait speed is a combination of stride length and cadence [36]. In the present study, the number of steps related to stride length was used. For the 10MWT, all patients walked without HAL assistance on a flat surface at a comfortable self-selected walking speed. They started walking before the starting line for the 10-m distance to accelerate and attain a stable speed before the test. To calculate walking speed, the walking time for 10 m was measured using a stopwatch. The number of steps was also counted for 10 m. During the measurement, the patients were allowed to use their assistive device and/or lower-limb orthosis as necessary. Therapists closely guarded the patients during the 10MWT against falls, for example, but did not provide physical assistance. Patients were required to use the same device and/or orthosis at the pre- and post-intervention measurements. The patients performed the 10MWT twice at each measurement. The best time of the two trials was used in the analysis [31]. Cadence was calculated as the number of steps divided by the walking time (steps/min).

The Berg balance scale (BBS) for balance function and 3-m timed up and go (TUG) test were determined before and after the intervention as other primary outcomes. For the BBS, 14 functional tasks were rated on a 5-point scale from 0 (lowest level of function) to 4 (highest level of function) by a therapist. The total scores ranged from 0 (severely impaired balance) to 56 (excellent balance). For the TUG, patients stood up from an armchair, walked 3 m, returned to the chair, and sat down using walking aids as required. This test assesses the patient’s dynamic balance ability and is a reliable and valid measure for stroke patients [37]. The best time of two trials was used in the analysis [38].

### Statistical analyses

Data are shown as means ± SD. To evaluate the feasibility of locomotor training using the HAL, the outcome measures were compared between pre- and post-training using a paired Wilcoxon’s test. The level of statistical significance for all measures was set at *P* < 0.05. The effect size (Cohen’s d) was calculated as the mean difference divided by the SD. A d value of 0.2 is considered a small effect size, 0.5 a medium effect size, and 0.8 a large effect size [39]. All statistical analyses were performed using SPSS software version 17.0 (SPSS Inc., Chicago, IL, USA).

## Results

All of the patients completed all 16 sessions. The CVC was used during locomotor training with the HAL in most patients. The CAC was used for the left knee joint in Case 4 between session 8 and session 12, because the motion provided by the CVC was slightly awkward for the patient. No training-related serious adverse events were observed.

The mean duration of the intervention period was 10.8 ± 3.5 weeks in all patients. The patient in Case 5 took 21 weeks to complete the intervention for his personal reasons, and his data were excluded from the analyses because of the deviation from the protocol. The patient in Case 4 needed to rest for 1 month after session 15 because of knee pain (patellar tendinitis); however, she completed the final session and her data were included in the analyses. Therefore, the 10MWT and BBS were evaluated in 15 patients, whereas the TUG test was evaluated in 14 patients. The patient in Case 7 experienced a physical burden, which was not able to tolerable for the TUG test.

The mean differences in walking speed, cadence, and number of steps for the group as a whole were 0.04 ± 0.11 m/s (P = 0.031, d = 0.34), 3.3 ± 13.3 steps/min (P = 0.017, d = 0.25) and −4.4 ± 8.0 steps (P = 0.020, d = 0.55), respectively (Table 2). The mean changes in BBS and TUG were 4.8 ± 7.0 (P = 0.004, d = 0.68) and −1.0 ± 4.7 s (P = 0.551, d = 0.22), respectively (Table 2).

**Table 2:** Comparison of outcomes between the start and end of the Hybrid Assistive Limb-assisted training program for the group as a whole

**Outcome measurements**		**Before training**	**After training**	**N**
10MWT	Speed (m/s)	0.41 ± 0.26	0.45 ± 0.24*	15
	Cadence (steps/min)	68.6 ± 26.4	72.0 ± 20.1*	15
	Number of steps (steps)	37.5 ± 22.7	33.1 ± 20.0*	15
BBS		40.6 ± 13.6	45.4 ± 8.02*	15
TUG (s)		36.0 ± 30.9	34.9 ± 29.5	14

Values are means ± standard deviation.

*Pre-post difference, P < 0.05.

*10MWT* 10-m walk test, *TUG* Timed-up and go. *BBS* Berg balance scale;co.

For the dependent ambulatory subgroup, the mean differences in walking speed, cadence, and number of steps were 0.07 ± 0.04 m/s (P = 0.012, d = 1.65), 7.5 ± 5.9 steps/min (P = 0.017, d = 1.26) and −8.0 ± 9.7 steps (P = 0.027, d = 0.82), respectively (Table 3). The mean changes in BBS and TUG were 7.0 ± 9.0 (P = 0.034, d = 0.78), and −2.9 ± 5.2 s (P = 0.237, d = 0.56), respectively (Table 3). For the independent ambulatory subgroup, the mean differences in walking speed, cadence, and number of steps were 0.005 ± 0.15 m/s (P = 0.612, d = 0.004), -1.4 ± 18.0 steps/min (P = 0.237, d = 0.08) and −0.29 ± 1.8 steps (P = 0.671, d = 0.16), respectively (Table 3). The mean changes in BBS and TUG were 2.3 ± 2.4 (P = 0.050, d = 0.94) and 0.9 ± 3.9 s (P = 0.398, d = 0.26), respectively (Table 3).

**Table 3:** Comparison of outcomes between the start and end of the Hybrid Assistive Limb-assisted training program for the subgroups

**Outcome measurements**		**Dependent ambulator**			**Independent ambulator**		
		**Before training**	**After training**	**n**	**Before training**	**After training**	**n**
10MWT	Speed (m/s)	0.24 ± 0.16	0.30 ± 0.19*	8	0.60 ± 0.21	0.60 ± 0.19	7
	Cadence (steps/min)	52.9 ± 14.4	60.4 ± 16.4*	8	86.5 ± 26.1	85.1 ± 18.2	7
	Number of steps (steps)	48.9 ± 26.6	40.9 ± 18.8*	8	24.4 ± 2.88	24.1 ± 3.18	7
BBS		33.6 ± 15.6	40.6 ± 8.07*	8	48.6 ± 3.31	50.9 ± 3.02*	7
TUG (s)		53.4 ± 35.8	50.5 ± 35.5	7	18.5 ± 8.58	19.4 ± 7.52	7

Values are means ± standard deviation.

*Pre-post difference, P < 0.05.

*10MWT* 10-m Walk Test, *TUG* Timed-up and go. *BBS* Berg balance scale.

## Discussion

The aim of the present study was to investigate the feasibility of locomotor training using the HAL for improving walking ability and balance in chronic stroke patients. The intervention significantly improved gait speed, cadence, and the number of steps assessed by the 10MWT as well as the BBS score. The TUG time also improved, although this difference was not statistically significant. The results obtained in this pilot clinical trial suggest that a HAL walking training program can improve walking speed, mainly in terms of both of cadence and the number of steps, and improve the balance ability on the basis of BBS.

To further investigate our findings on the feasibility of locomotor training using the HAL, we explored how the subgroups based on functional ambulation influenced the results. The dependent ambulatory subgroup displayed significant differences in walking speed, cadence, and number of steps between before and after the HAL training, with large effect sizes (d > 0.8). In contrast, these differences were not significant for the independent ambulatory subgroup. Although the difference in BBS score was statistically significant in both subgroups, the effect size in the dependent ambulatory subgroup (d > 0.8) was lager than in the independent ambulatory subgroup (d < 0.3). These findings indicate that the differences in walking speed, cadence, and number of steps of the group as a whole was primarily because of those in the dependent ambulatory subgroup.

Several studies have investigated the effects of locomotor training using robots for chronic stroke patients. Using the Lokomat, Hornby et al. [22] reported a 0.07 m/s increase (d = 1.0) in gait speed from the mean baseline value of 0.45 m/s. Similarly, Westlake & Patten [25] reported a 0.1-m/s increase (d = 0.32) from the mean baseline value of 0.62 m/s. Using other robots, Peurala et al. reported a mean increase in walking speed of 0.08 m/s from the mean baseline value of 0.25 m/s with the Gait Trainer alone and a 0.05 m/s increase from 0.23 m/s at baseline using the Gait Trainer with functional electrical stimulation [28]. It is difficult to compare these studies with the present study because of differences in baseline walking ability and study settings. However, the increase in walking speed after the HAL training program in the dependent ambulatory subgroup (0.07-m/s increase from the mean baseline of 0.24 m/s, d = 1.64) is consistent with the result of locomotor training with other robots.

In recent years, a top-down approach has emerged as a new rehabilitative methodology. Belda-Lois et al. defined this approach as rehabilitation therapies based on the state of the brain instead of the bottom-up approach, which acts on the physical level [40]. The top-down approach is considered highly promising from the viewpoint of neurorehabilitation because it promotes neuroplasticity. This approach is mainly applied in functional electrical stimulation, assistive robotic devices, and brain–computer interfaces that use myoelectric or brain activity during the patient’s volitional control. Locomotor training using the HAL is based on the top-down approach. The HAL assists motion by myoelectric activity on the basis of the patient’s voluntary drive. The voluntary drive and thus the motion normalized by the assistance provided by the external device forms the foundation for a proprioceptive feedback loop for patients with lesions involving the sensory pathways. The neural activity associated with voluntary drive and normalized motion while repeatedly and intensively executing specific tasks promotes learning [41] and then leads to the reinstatement or restructuring of appropriate proprioceptive feedback. This mechanism explains the therapeutic effect of locomotor training using HAL as one of these top-down approaches.

Some robots have been used for active training during walking rehabilitation. The Lokomat autonomously provides a predefined walking motion that is not related to the user’s voluntary drive. The user generates force along the autonomous motion pattern generated by the Lokomat and is encouraged to generate as much effort as possible [20-23]. In contrast, the HAL generates assistive torque according to the amount of the bioelectric signals generated the user’s voluntary muscle activities. The user obtains motion assistance while simultaneously operating HAL on the basis of the user’s voluntary drive [32]. Therefore, the user is able to control the amount of assistance provided by HAL by voluntarily adjusting their myoelectric activities. As mentioned above, this mechanism forms a proprioceptive feedback loop. A visual feedback loop would be also formed because the patients are able to directly observe the supported motion. Therefore, the user makes effort to produce the interactive motion as close as possible to the motion pattern of normal gait by using the perceptual feedback. This process might improve the gait patterns of patients with chronic stroke. As a result, the locomotor performance differed significantly before and after the HAL training. To our knowledge, the present study was the first to investigate the feasibility of the locomotor training with the interactive motion provided by the HAL and under voluntary user control.

Randomized controlled trials are now needed to confirm the efficacy of HAL-assisted training compared with conventional physical therapy. Studies are also needed to examine the characteristics of patients who experience the greatest benefits with HAL-assisted locomotor training by examining features such as walking ability or severity of paralysis at baseline. Studies should also determine the optimal frequency and duration of the HAL-assisted training. Information analysis on these studies are essential to develop the most effective operating procedures to enable therapists to adjust the amount of interactive motion provided by the HAL according to the user’s voluntary drive.

## Conclusions

In this study, we confirmed the feasibility of HAL-assisted locomotor training for chronic stroke patients. It is interesting to note that the findings in the dependent ambulatory subgroup primarily contributed to the significant differences. This study is the first step toward demonstrating the clinical potential of locomotor training with a robot providing interactive motion based on voluntary drive. The next step would be randomized clinical studies to compare the efficacy of HAL-assisted training with that of conventional therapies in terms of improving walking ability in patients with chronic stroke and to develop effective operating procedures for using the HAL.

## Abbreviations

HAL: Hybrid assistive limb; CVC: Cybernic voluntary control; CAC: Cybernic autonomous control; 10MWT: 10-meter walk test; TUG: Timed up and go; BBS: Berg balance scale.

## Competing interests

HK is a founder, shareholder, and an external director of CYBERDYNE Inc. which produces the HAL. YS is a founder, shareholder, and the CEO of CYBERDYNE Inc. CYBERDYNE was not involved in study design, data collection, analysis, or writing or submission of this article.

## Authors’ contributions

HK administered the intervention, performed statistical analysis and drafted the manuscript. KK, YS, MS, and NO helped to design the study and draft the manuscript. YN supported the statistical analysis and helped to draft the manuscript. KY and RA administered the intervention and helped collect data. KE is the principal investigator of this study, and helped to design and coordinate the study. All authors read and approved the final manuscript.

## Pre-publication history

The pre-publication history for this paper can be accessed here:

http://www.biomedcentral.com/1471-2377/13/141/prepub
